# Speech Outcomes After Secondary Furlow Z-Plasty and Pharyngeal Flap Procedure

**DOI:** 10.1097/SCS.0000000000011465

**Published:** 2025-05-24

**Authors:** Anika Szwedyc, Suvi Alaluusua, Pia Vuola, Veera Pitkänen, Anne Saarikko

**Affiliations:** Cleft and Craniofacial Center, Helsinki University Hospital, Helsinki, Finland

**Keywords:** Furlow Z-plasty, pharyngeal flap, velopharyngeal function, velopharyngeal insufficiency

## Abstract

The optimal surgical approach for treating velopharyngeal insufficiency (VPI) in patients with cleft is unknown. Hence, the authors reported the outcomes and success rates of the Furlow Z-plasty and pharyngeal flap procedures for treating VPI in all types of clefts. The authors included 377 patients in this study, of whom 351 and 26 underwent Furlow Z-plasty and the pharyngeal flap procedures, respectively. The authors retrospectively assessed the outcomes of both procedures in 3 groups: the nonsyndromic, Robin sequence (RS), and syndromic groups. All patients were evaluated by speech pathologists preoperatively and postoperatively. The success rate of VPI correction after Furlow Z-plasty was 82%; however, 12% required a reoperation for residual VPI. Patients in the syndromic group had significantly higher VPI rates than their nonsyndromic counterparts (*P*<0.001). The success rate was 81% after the pharyngeal flap procedure; however, 8% of patients required a second operation for residual VPI. The postoperative VPF of patients in the nonsyndromic and RS groups did not significantly differ compared with the syndromic group (*P*=0.782). Some patients (12%) developed obstructive sleep apnea (OSA) after the pharyngeal flap procedure; however, none developed it after the Furlow Z-plasty. The median hospital length of stay after the pharyngeal flap procedure was prolonged compared with that after the Furlow Z-plasty (*P*<0.001). Both procedures were effective in correcting VPI. Children in the syndromic group had an increased rate of VPI after Furlow Z-plasty. Development of OSA and prolonged hospital stay were observed after the pharyngeal flap procedure.

Orofacial clefts are among the most common congenital defects, occurring in ~1 in 700 to 1500 births globally.^1–3^ However, prevalence rates vary significantly by geographic region, ethnicity, sex, and environmental factors.^4,5^ After primary palatoplasty, the inability to completely close the velopharyngeal port during speech can result in velopharyngeal insufficiency (VPI). Assessing children with cleft palate and VPI is crucial in ensuring successful outcomes of their surgical treatment. The rate of VPI surgery after primary palatoplasty varies based on the surgical technique, surgeon experience, and if the patient has a syndrome or Robin sequence (RS).^6–9^

On the basis of the approach used, VPI in patients with cleft palate is categorized as either static or functional. Static approaches, including pharyngeal flap and sphincter pharyngoplasty, obstruct the pharynx and facilitate velopharyngeal closure. In contrast, functional approaches are used to rebuild the pharyngeal ring through techniques such as palatal muscle repair and lengthening, with or without double-opposing Z-plasty.^10,11^ In mild cases of VPI, posterior wall augmentation may be a less invasive alternative.^12,13^ However, findings from some studies reveal that posterior wall augmentation may have unpredictable outcomes.^14^ There have been several attempts to identify the optimal procedure for VPI; however, a definitive solution has not yet been established.^15,16^ A single surgical method may not resolve all cases of VPI owing to several underlying medical conditions, cognitive abilities, differing primary cleft palate closure techniques, and the frequency of previous surgeries. Successful outcomes are often achieved by tailoring the procedure to each patient’s needs using early identification, comprehensive preoperative evaluation, and management by an experienced multidisciplinary team. The focus of the preoperative evaluation is on assessing velopharyngeal function (VPF) using clinical evaluation, such as auditory perceptual judgment, which can be supplemented using an instrumental analysis of the velopharyngeal valve.^17^

The pharyngeal flap procedure is one of the oldest and most widely used surgical techniques and is considered one of the most effective methods for treating VPI.^18–20^ In our experience, patients with good lateral wall motion or those who are syndromic with a motionless palate may benefit from the superiorly based pharyngeal flap procedure. This procedure has high success rates; however, a notable drawback is the risk of developing obstructive sleep apnea (OSA).^19,21–24^ The primary advantage of Furlow Z-plasty is that it enables the desired VPF through palatal lengthening without compromising the upper airway.^24,25^ This approach may be particularly beneficial for patients with primary straight-line closure of the palate, limited lateral wall motion, or those at high risk of developing airway obstruction.

Successful VPI surgery involves evaluation using comprehensive measures that address both functional and standardized outcomes. In this study, we aimed to assess the outcomes of patients who underwent VPI surgery, specifically Furlow Z-plasty and the pharyngeal flap procedure, for nonsyndromic, RS, and syndromic cleft palate.

## MATERIALS AND METHODS

### Participants

**FIGURE 1:**
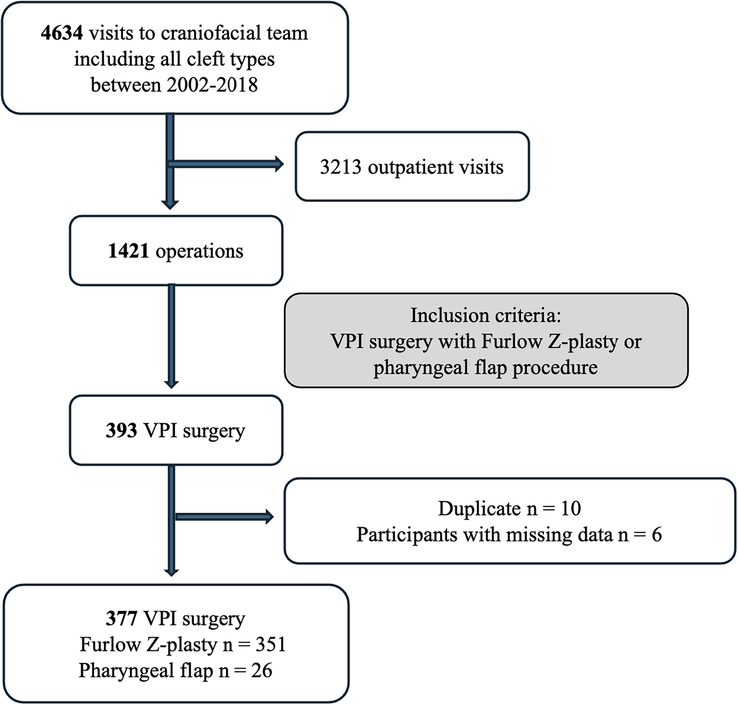
Study cohort flow chart.

The study was approved by the University Hospital Research Committee following the principles outlined in the Declaration of Helsinki. Data for this study were obtained from the University Hospital Patient Database, which was supplied anonymously. We included 4634 outpatients and inpatients who were treated by a multidisciplinary craniofacial team at a single center between 2002 and 2018 (Fig. 1). We found 393 VPI surgeries that were performed during this period. The participants who underwent these surgeries were born between the years 1993 and 2013. After excluding 10 duplicates and 6 cases with missing data, 377 patients met the inclusion criteria for treatment using either the Furlow Z-plasty or pharyngeal flap procedure for VPI. Comprehensive medical and developmental histories were documented. In addition, all patients underwent preoperative and postoperative speech evaluations at the same center.

The craniofacial team performing the VPI surgeries included 5 cleft surgeons, who operated on all types of cleft: bilateral cleft lip and palate, unilateral cleft lip and palate, isolated cleft palate, and submucous cleft palate. Furthermore, data on primary palatoplasty methods were collected from the same center (Supplementary Table 1, Supplemental Digital Content 1, http://links.lww.com/SCS/H847). However, information was unavailable for 49 patients because they underwent their primary surgery at another center.

The patients were divided into 2 groups based on the type of VPI procedure they underwent. The imbalance observed between the 2 groups was because the Furlow Z-plasty method was preferred during the study period. Subsequently, the results of each group were retrospectively analyzed in patients who were nonsyndromic, RS, and syndromic by comparing the preoperative and postoperative results of each group. Outcomes were compared between groups.

### Velopharyngeal Insufficiency Surgery

The decision to perform VPI surgery was made based on perceptual and instrumental assessments. The instrumental assessment included case-based evaluation of nasometry with or without videofluoroscopy and/or videonasoendoscopy. Teams of plastic surgeons, speech pathologists, and/or phoniatricians conducted a preoperative evaluation together to determine whether to operate on the VPI using Furlow Z-plasty or pharyngeal flap procedure.

### Pharyngeal Flap

The length of the palatal defect was evaluated and measured during the procedure. An incision plan was outlined on the posterior wall of the pharynx. Subsequently, a superior-based pharyngeal myomucosal flap was carefully raised from the posterior wall of the pharynx, and the lower end of the flap was gently shaped into a V. The flap width was determined by considering the distance to the lateral pharyngeal wall, whereas the length was modified to cover the palatal defect and fit the soft palate completely. A tunnel resembling the shape of a fish mouth was formed along the posterior free edge of the palate. This was followed by inserting the free end of the flap into the tunnel, with its uncovered end passing through and carefully pulled deeply between the oral and nasal layers of the soft palate. Monocryl sutures (4-0) were used to advance the free edge of the flap through the tunnel, followed by a second layer of suturing at the back, which connected the edge of the palatal defect in the soft palate to the flap. This resulted in a binomial nasopharyngeal appearance. Subsequently, the donor area of the pharynx was closed using the Monocryl sutures. However, if any tension was observed, the donor area was left partially open to allow for healing through granulation. Finally, lidocaine with adrenaline was administered to achieve hemostasis at the end of the procedure.

### Furlow Z-Plasty Procedure

During the procedure, the incision lines were marked on the oral mucosa according to the typical Furlow Z-plasty pattern, which includes the anterior and posterior limbs.^26^ The final incisions were made after the flap was freed, ensuring a tension-free wound closure under loupe magnification or microscopy, as determined by the surgeon. Initially, the incision was made in the oral mucosa. Subsequently, a complete midline incision was made through the palatal mucosa. A myomucosal flap was created from the nasal layer on the left, whereas the levator muscle remained attached to the oral mucosa. No muscle fibers were attached to the nasal mucosa at the levator muscle. Next, an incision was made on the right side, and the oral mucosal flap was elevated, ensuring that all muscle fibers were attached to the nasal layer. The Z-plasty flaps were created on the nasal layer in a mirror-image pattern. The levator muscle was repositioned using a nasal Z-plasty flap on the right side of the soft palate, whereas, on the left side, the nasal mucosal flap was anteriorly rotated. Consequently, the nasal flaps were exchanged using a Z-plasty configuration, and the mucosal closure was completed using 5-0 Monocryl sutures. The levator muscle ring was initially reconstructed using either 4-0 Monocryl or 4-0 polydioxanone sutures. The oral layer was closed using a Z-plasty technique, which involved transposing the flaps, using 4-0 and 5-0 Monocryl sutures. Lidocaine with adrenaline was administered to achieve hemostasis at the end of the procedure.

### Speech Assessment

The children were evaluated preoperatively and postoperatively during the multidisciplinary cleft team visits at the same center. A speech pathologist evaluated the VPF at both time points. In the final preoperative and postoperative speech assessments, the patient’s speech records were analyzed in a blinded manner. The rating was assessed using all speech materials, including a single-word list, sentence repetition, and a sample of connected speech. An overall assessment of the symptoms of VPI, including hypernasality, audible air leakage, weakness in pressure consonants, and non-oral articulation errors, was conducted using the rating scale for velopharyngeal competence (VPC-R) (0=competent, 1=marginally incompetent, 2=incompetent). The VPC-R classification is a reliable and validated method for the overall assessment of velopharyngeal function.^27,28^ The VPC-R is described in detail in Supplementary Table 2, Supplemental Digital Content 1, http://links.lww.com/SCS/H847.

Nasoendoscopy and videofluoroscopy are frequently required in strategizing VPI surgery; however, neither procedure is typically used in evaluating VPI. Nasoendoscopy is used to produce images axially, with enhanced difficulty in interpreting the pharyngeal wall movement.^29,30^ We recently enhanced our preoperative examination protocol by incorporating routine nasoendoscopy. This approach enables a more personalized and precise evaluation, including the palatal muscles orientation, dynamics of palatal motion, and measurement of the velopharyngeal (VP) gap size in the future.

Statistical analyses were performed preoperatively and postoperatively using VPC-R. The success rate and adequate VPF were defined by VPC-R scores of 0 and 1, indicating normal or sufficient postoperative VP closure. An improvement in VPF was noted when the postoperative score was <2. The first postoperative follow-up visit was performed between 6 and 12 months. All the patients included in this study were followed up for at least 48 months.

### Additional Outcome Measures

Additional outcome measures, including postoperative complications, reoperation rates, and length of stay (LOS) in the hospital, were documented. These outcome measures were obtained from patient records, which also included data on OSA and whether polysomnography (PSG) was performed.

### Statistical Analysis

Data were analyzed using SPSS version 29 (IBM Corp.). The Kruskal-Wallis test was used to examine the effect on speech outcomes in all 3 groups that underwent VPI surgery using Furlow Z-plasty. The speech results were measured on an ordinal scale, hence nonparametric tests were used for the analysis. All significant tests used were 2-tailed. The groups were examined as separate samples to account for possible confounding. The Mann-Whitney *U* test was used to determine whether the effect of the pharyngeal flap procedure on speech results differed between the nonsyndromic and syndromic groups. Furthermore, it was used to detect any differences in the LOS between both procedures. LOS was found to be non-normally distributed, as indicated by the Q-Q plot, Kolmogorov-Smirnov test, and Shapiro-Wilk test. The Wilcoxon signed-rank test was used to compare the pre- and postoperative VPC ratings. Pearson χ^2^ and Fisher tests were used to compare success rates between different patient groups. The Bonferroni correction was applied to adjust the significance levels in pairwise comparisons of the 3 groups that underwent the Furlow Z-plasty. The χ^2^ test was used to identify any differences between the surgeons and the outcome measures. Statistical significance was set at *P*<0.05.

## RESULTS

### Patient Characteristics

The characteristics of the 377 patients, including all cleft types, observed in the 3 groups (nonsyndromic, RS, and syndromic) are shown in Supplementary Table 3, Supplemental Digital Content 1, http://links.lww.com/SCS/H847.

### Overall Outcomes and Improvement in Velopharyngeal Competence

**FIGURE 2:**
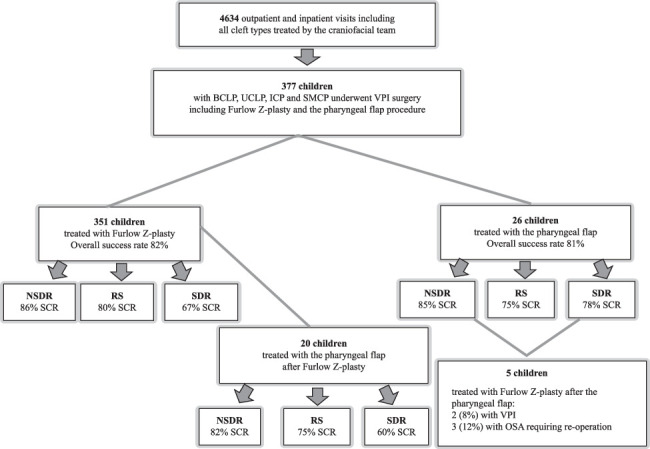
Study participants and overall outcomes.

We observed 351 patients (93%) who underwent Furlow Z-plasty and 26 (7%) who underwent a pharyngeal flap procedure (Fig. 2). All patients were preoperatively evaluated as incompetent by speech pathologists. VPF was improved, and an adequate VPF was achieved postoperatively in 289 (82%) patients after Furlow Z-plasty. However, VPF did not improve in 62 (18%) patients, and 41 (12%) of them required reoperation for residual VPI, of which 20 (49%) underwent a pharyngeal flap procedure. A statistically significant improvement was observed between the preoperative and postoperative VPC ratings (Z=−16.022, *P*<0.001). Postoperative VPF was improved, and an adequate VPF was accomplished in 21 (81%) patients after the pharyngeal flap procedure. Nevertheless, VPF did not improve in 5 (19%) patients, and 2 (8%) required reoperation for residual VPI with Furlow Z-plasty. The improvement shown between the preoperative and postoperative VPC ratings was statistically significant (Z=−4.583, *P*<0.001).

### Furlow Z-Plasty Outcomes in the 3 Groups and the Need for Secondary Velopharyngeal Insufficiency Surgery

**FIGURE 3:**
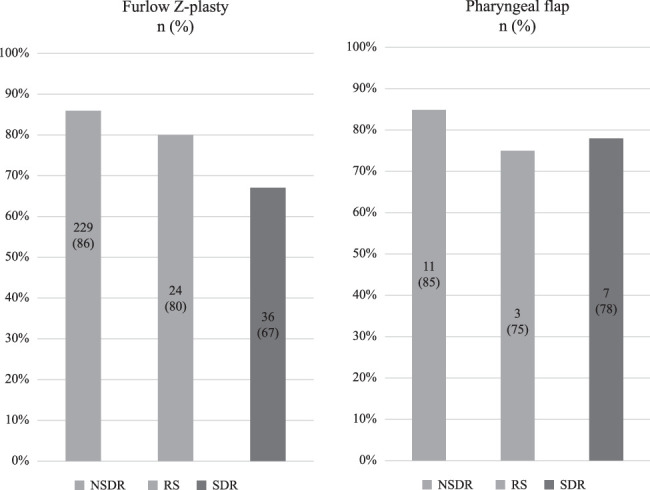
Success rates of 377 patients who underwent speech-correcting surgery.

Adequate VPF was achieved in 229, 24, and 36 (86, 80, and 67%, respectively) nonsyndromic, RS, and syndromic patients undergoing Furlow Z-plasty (Fig. 3). The success rates significantly differed between the 3 groups (χ (2)=11.391, *P*=0.003). Regarding the group comparisons, the postoperative speech outcome did not significantly differ between patients with a history of RS and nonsyndromic patients (χ [1]=0.711, *P*=0.399). Similarly, patients with a history of RS did not significantly differ from syndromic patients (χ [1]=1.680, *P*=0.197). Syndromic patients had a significantly higher rate of VPI than nonsyndromic patients (χ [1]=11.379, *P*<0.001). The operative effect on VPF between the 3 groups using the Kruskal-Wallis test was statistically significant (χ [2]=13.319, *P*=0.001).

The following patients underwent secondary VPI surgery after Furlow Z-plasty: 18 (5%) who had undergone a second Furlow Z-plasty, 20 (6%) who used pharyngeal flap procedures and 3 (1%) who had muscle-releasing procedures to enhance palate movement. Of the 20 patients who underwent secondary VPI surgery with the pharyngeal flap procedure, 11, 4, and 5 (55, 20, and 25%, respectively) were nonsyndromic, RS, and syndromic, respectively. Adequate VPF was achieved in 15 (75%) of these patients; however, the speech results did not improve in 5 (25%) of them. Adequate improvement was achieved in 9, 3, and 3 (82, 75, and 60%, respectively) nonsyndromic, RS, and syndromic patients, respectively, who underwent secondary VPI surgery with a pharyngeal flap procedure. Of the 18 patients who underwent secondary VPI surgery with Furlow Z-plasty, 13, 3, and 2 (72, 17, and 2%, respectively) were nonsyndromic, RS, and syndromic. Adequate VPF was achieved in 14 (78%) of these patients; however, speech results did not improve in 4 (22%). Furthermore, adequate VPF was achieved in 12, none, and 2 (67, 0, and 2%, respectively) of the nonsyndromic RS and in the 2 syndromic patients who underwent secondary VPI surgery with Furlow Z-plasty. One syndromic patient underwent a muscle-releasing procedure, after which adequate VPF was achieved following Furlow Z-plasty. We chose not to conduct hypothesis testing owing to the small sample size.

### Pharyngeal Flap Procedure Outcomes in 3 Groups and the Need for Secondary VPI Surgery

Adequate VPF was achieved in 11, 3, and 7 (85, 75, and 78%, respectively) nonsyndromic, RS, and syndromic patients undergoing the pharyngeal flap (Fig. 3). The postoperative speech outcomes did not significantly differ between the nonsyndromic and syndromic patients (U=73.000, *P*=0.782). Notably, we did not perform statistical tests to compare patients with a history of RS because of the small number of patients with clefts in the group.

The need for a secondary VPI surgery with Furlow Z-plasty was recorded for 2 (8%) patients after the pharyngeal flap procedure, and neither patient achieved speech improvement after reoperation.

### Obstructive Sleep Apnea

Five (1%) patients developed mild obstruction after Furlow Z-plasty, which was diagnosed using PSG, requiring no further intervention. Therefore, positional guidance alone was recommended for these patients. In comparison, 1 patient developed mild OSA (4%), and 3 (12%) patients developed OSA that required further surgical intervention after the pharyngeal flap procedure. Excluding patients with mild obstruction, all others required further intervention. The pharyngeal flap was cut, and reoperation was performed using Furlow Z-plasty. At the time of the pharyngeal flap surgeries, the primary goal was to achieve the best possible speech outcomes. This focus may have resulted in the use of a wider flap design, which could have contributed to the development of OSA after the pharyngeal flap. However, we have shifted towards a more tailored approach, over the past decade.

### Postoperative Complications

Postoperative hematoma and mild wound dehiscence were observed in 10 (3%) patients after Furlow Z-plasty. Seven patients had mild wound dehiscence, which had completely healed by the time of the postoperative follow-up visit. Of these, 2 had a small fistula in the posterior part of the palate, which had healed at the 6-month postoperative follow-up. Three patients developed immediate postoperative hematomas in which primary wound closure was reperformed. One episode of postoperative infection was observed and treated with a 1-week course of oral antibiotics.

One patient (4%) had mild wound dehiscence, which healed within 1 week postoperatively, after the pharyngeal flap procedure.

### Length of Stay in Hospital

**FIGURE 4:**
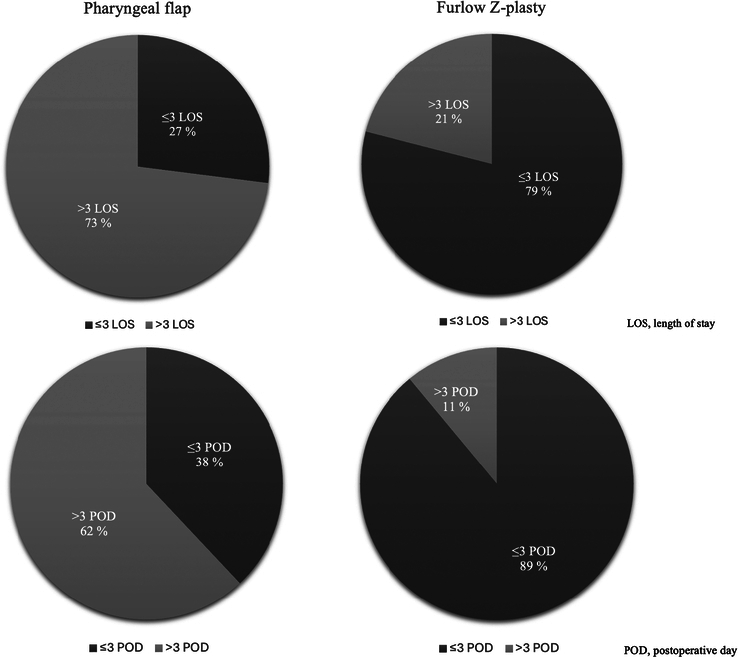
Pie chart demonstrating the length of stay in hospital.

The LOS of patients in the hospital from admission to discharge is presented along with the postoperative days (POD) after surgery in Fig. 4. Owing to our centralized cleft care, patients often travel long distances (up to 1100 km) to reach our hospital, and in the past, families may have come to the hospital even days before their surgery. Recognizing this, we used a comprehensive postoperative discharge protocol to ensure optimal care and a safe transition back home. After surgery, patients meet with the cleft team, which includes the operating surgeon, nurses, and speech pathologist, for a thorough follow-up. Discharge is considered when patients can swallow food, are pain-free without the need for strong pain medication, and their families are able to manage daily challenges at home with clear postoperative instructions. In this study, the LOS is defined as the number of PODs. The median hospital LOS after Furlow Z-plasty and the pharyngeal flap procedure was 2 and 4 (IQR, 2–3 and 3–4, respectively) days, respectively (*P*<0.001). Most patients undergoing Furlow Z-plasty who had 3 or more PODs in the hospital underwent concomitant operations such as lip remodeling, alar remodeling, rhinoplasty, or re-tympanostomy in the same session. Patients who underwent the pharyngeal flap procedure and stayed in the hospital longer than 4 PODs were often observed to have pain, feeding difficulties, or a prolonged stay in the intensive care unit due to obstruction. However, over the past decade, the LOS at our center has become shorter compared with the results presented in this study.

### Surgeons and Outcome Measures

An inevitable potential for bias is introduced when surgeries are performed by 5 different surgeons. This is because variations in surgical experience, preferences, interoperative management strategies, care planning, and levels of attentiveness may have influenced the outcome measures. In this study, the differences in success rates, LOS, complications, and reoperations between surgeons were not statistically significant (*P*<0.05). A critical interpretation of these results is essential due to the existing disparities.

## DISCUSSION

In this study, among the 377 patients who underwent VPI surgery, 82% achieved adequate VPF after Furlow Z-plasty, whereas 81% achieved it after the pharyngeal flap procedure. Patients who were syndromic achieved adequate VPF less frequently (67%) than those who were nonsyndromic (86%) after Furlow Z-plasty. Speech outcomes were similar for patients who were nonsyndromic and those with a history of RS (80%). Adequate VPF was less frequently achieved in patients who were syndromic (78%) compared with those who were not (85%) after the pharyngeal flap procedure. No cases of OSA were found after Furlow Z-plasty; however, after the pharyngeal flap procedure, 12% of patients developed OSA requiring resection of the pharyngeal flap. The need for secondary VPI surgery was low, occurring in 12% of patients after Furlow Z-plasty and 8% after the pharyngeal flap procedures. In addition, the pharyngeal flap procedure was associated with a prolonged hospital stay of ≥4 days.

VPI occurs in 5% to 86% of patients after cleft palate repair.^20,23,31–33^ Most surgeons use their preferred procedure, with the pharyngeal flap procedure being the most commonly used, with a reported success rate of 81% to 97%.^20,34–38^ In our study, the overall success rate after the pharyngeal flap procedure was 81%, which is comparable to previous studies. However, speech outcomes did not significantly differ between patients with and without syndromes. Notably, the pharyngeal flap procedure was initially introduced by Passavant and Schoenborn,^39,40^ and has undergone several modifications. At our center, the preferred procedure involves displacing the flap posterosuperiorly into the oropharynx and soft palate, as described by C. A Honig and V.M. Hogan.^41,42^ Customizing the type of pharyngoplasty using the shape of the defect observed during nasoendoscopy has been suggested; however, evidence supporting this approach is limited.^43^

Despite careful planning and surgery, complications may arise, with OSA being the most common. We observed OSA in 12% of patients. Meanwhile, previous studies have reported incidences between 0% and 96% after the pharyngeal flap procedure.^25,44–47^ There are several factors behind this disparity in outcomes, including patient population differences, variations in pharyngeal flap techniques, and differences in the care provided. In a literature review, it was indicated that minor obstruction, hyponasal speech, and snoring are common after the pharyngeal flap procedure but tend to relatively decrease several months postoperatively.^46,48^ Predictors of OSA before surgery may include patients who are syndromic, those with a history of RS, those with structural narrowing of the upper airway, and those with enlarged tonsils.^49–54^ This aligns with our study, in which 60% of patients diagnosed postoperatively with OSA were syndromic, of which one was syndromic with a history of RS. However, at the time of the pharyngeal flap surgeries, we could not perform routine preoperative endoscopy. Furthermore, intraoperative planning was based on clinical assessment of flap design, with the primary goal of achieving the best possible speech outcomes. This may have involved a wider flap design, which could have contributed to the development of OSA observed in this study. Severe OSA may require pharyngeal flap dissection, often requiring an alternative surgery for VPI. De Blacam et al showed that with a midline pharyngeal flap, the need for flap dissection due to OSA occurred in 3.6% of cases.^55^ All patients diagnosed with OSA in our study required pharyngeal flap dissection, which resulted in a Furlow Z-plasty intraoperatively. Robinson et al^56^ in their study, reported an ~3 times higher incidence of OSA in children with cleft palate than in those without cleft. Globally, more than 400 million people have at least moderate OSA^57^ and there has been a strong prevalence of OSA among adults (14%–50%) in the past 2 decades.^57–59^ Nonetheless, future studies with long-term follow-up are needed to evaluate the prevalence of OSA in children with cleft palate.

Leonard Furlow described the Furlow Z-plasty,^26^ which is widely used and associated with low occurrence of OSA development postoperatively.^45,60^ Furlow Z-plasty involves reconstructing the levator sling by displacing the levator veli palatini muscles so they overlap. This configuration is believed to improve the approximation of the natural sutures in the oral mucosa, thereby reducing patients’ predisposition to OSA during the pharyngeal flap procedure, in which the flap is locked in the posterior wall of the oropharynx.^25,61–63^ Our experience with Furlow Z-plasty includes treating all grades of VPI, even when a large velopharyngeal gap is present. In this study, we present an overall success rate of 82% for Furlow Z-plasty, which is in line with earlier studies that show the success rates were up to 97%.^64–71^ A significant advantage of this procedure is the low risk of postoperative OSA. In our study population, 1% had a positive record of mild obstruction on polysomnography, and none developed OSA. In addition, no surgical interventions were performed; however, positional guidance was provided. Postoperative persistent VPI is often observed in ~17% of cases, which is consistent with our results of 12%.^65,72^ Persistent VPI led to a second VPI surgery, with an overall success rate of 75% achieved for the pharyngeal flap procedure in this study. Our experience has convincingly shown that a second surgery with Furlow Z-plasty after a pharyngeal flap procedure is more simplified than the opposite; however, this does not apply if the pharyngeal flap was initially resected owing to OSA.

Some cleft centers reported that the success rates of VPI surgery did not differ between syndromic and nonsyndromic patients.^73^ Findings from the literature review showed that patients with syndromes had lower success rates in VPI surgery than nonsyndromic patients, especially if the 22q11.2 deletion syndrome was present, leading to a more residual VPI.^8,73,74^ Seven patients had 22q11.2 deletion, of whom 6 and 1 underwent Furlow Z-plasty and pharyngeal flap procedure, respectively. VPF improved in 4 and after Furlow Z-plasty, whereas only one patient had improved function after the pharyngeal flap procedure. Moreover, 2 patients had residual VPI after Furlow Z-plasty. In a recent meta-analysis, it was reported that higher revision rates and a lower likelihood of achieving normal resonance were found after the primary surgery in children with syndromes compared with those without.^71^ We discovered increased success rates in the nonsyndromic group: 86% and 85%, after Furlow Z-plasty and the pharyngeal flap procedures, respectively. In contrast, lower success rates were observed in patients with syndromes: 67% and 78% after Furlow Z-plasty and the pharyngeal flap procedure, respectively. In both procedures, there were lower success rates in syndromic patients; however, the statistically significantly lower rates were associated with the Furlow Z-plasty approach. To the best of our knowledge, the speech outcomes of patients with the syndrome who underwent these 2 surgical procedures have not been previously reported.

RS is classically described as the clinical triad of micrognathia, glossoptosis, and upper airway obstruction.^75^ A history of RS might be associated with the syndromes.^76^ The higher incidences of VPI and VPI-related surgeries in patients with a history of RS remains unclear.^9,77^ The success rates reported for these patients were 40% to 79% after Furlow Z-plasty and 80% after the pharyngeal flap.^65,78^ Both findings were comparable to our results of 80% and 75% success rates in nonsyndromic patients with a history of RS after Furlow Z-plasty and pharyngeal flap procedures.

The LOS is often influenced by factors including treatment center accessibility, concomitant surgeries, feeding difficulties, postoperative pain, and emergent postoperative complications such as obstruction, bleeding, or infection. Furthermore, multivariate predictors of LOS include increasing patient age, male sex, lack of syndromic association, intraoperative antiemetics and steroids, and shorter anesthesia duration.^79^ Globally, hospital stays after pediatric and cleft surgery have reduced.^80,81^ In a previous study, patients who underwent the pharyngeal flap procedure for VPI often required additional support or intervention in the postoperative period; hence, early discharge was considered unsafe.^79^ In our study, a prolonged LOS after the pharyngeal flap was commonly associated with postoperative pain, feeding difficulties, and obstruction, with 62% of patients being discharged on the fourth postoperative day. Patients treated with Furlow Z-plasty (89%) were discharged at least one day earlier, on the third postoperative day, in the present study.

The strength of this study lies in the relatively large study group of all cleft types obtained at a single center, where patients received regular care in a multidisciplinary setting. However, there were some limitations in this study. First, the retrospective design poses an inherent bias risk. Second, the lower quality of the medical records may have led to errors in data interpretation and evaluations of the surgical techniques. Surgeons tailor their postoperative care or techniques to accommodate several patient anatomies during the procedure, which is standard practice in surgery. All procedures included in the study were performed by 3 experienced cleft surgeons and 2 junior team members, which possibly introduced a potential bias. PSG was performed only in children presenting with obstructive symptoms, which may have led to undetected cases of mild OSA in some children. An additional limitation of this study was the absence of patient-reported outcomes.

There results of this study show that Furlow Z-plasty might be as effective for correcting VPI as the pharyngeal flap procedure, with fewer adverse effects, such as OSA. Nevertheless, there is a potential for selection bias, limiting the generalizability of the findings to different populations. In this study, it is recommended that all patients with a history of RS or other syndromes undergo PSG preoperatively before planning VPI surgery using the pharyngeal flap procedure, even in a secondary VPI surgery setting.^82^ Furlow Z-plasty is often preferred over the pharyngeal flap procedure in cases of mild obstruction detected preoperatively. However, the pharyngeal flap procedure may be more effective in patients with the syndrome. The findings of this study can guide professionals in selecting the most appropriate choice of VPI surgery for specific patient profiles. However, larger multicenter studies are needed to assess the extended effects of each procedure on the quality of life and speech function across diverse patient populations.

## CONCLUSION

The results of this study indicate that both VPI surgeries are effective, with a success rate of 82% for Furlow Z-plasty and 81% for the pharyngeal flap procedure. Furlow Z-plasty may not be effective in syndromic patients; however, it is rarely associated with obstruction or a prolonged hospital stay. Nevertheless, the focus of future research should be directed toward the long-term effects and outcomes of these surgeries across diverse populations.

## Supplementary Material

SUPPLEMENTARY MATERIAL
