# Supplementary material for: Speech Outcomes After Secondary Furlow Z-Plasty and Pharyngeal Flap Procedure
Source: J Craniofac Surg. 2025 May 24;36(8):2786–93. doi: 10.1097/SCS.0000000000011465 (PMC12537044; doi:10.1097/SCS.0000000000011465)
Supplement: SUPPLEMENTARY MATERIAL [file scs-36-02786-s001.docx]

**Supplementary Table 1. Primary palatoplasty of 325 patients**

| **Primary palatoplasty** | **No. of patients (%)** |
| --- | --- |
| **One-staged** | **193 (59)** |
| **Two-staged** | **83 (26)** |
| Hard palate* | 64 (20) |
| Soft palate* | 19 (6) |
| **Unknown** | **49 (15)** |

*At the first stage

**Supplementary Table 2. Rating Scale for velopharyngeal competence, VPC-R**

| **Score** | **Velopharyngeal closure** | **Comment** |
| --- | --- | --- |
| 0 | Competent | Competent velopharyngeal closure |
| 1 | Marginally competent | Evidence of minor problems suggesting borderline closure |
| 2 | Incompetent | Evidence of significant problems usually requiring surgical management |

Success rate (SCR) is defined by including scores 0 and 1 postoperatively.

**Supplementary Table 3. Characteristics of the 377 patients in this study**

**Furlow Z-plasty (n=351)**

|  | Nonsyndromic  (n=267) | RS  (n=30) | Syndromic    (n=54) |
| --- | --- | --- | --- |
| Male, n (%) | 136 (51) | 16 (53) | 28 (52) |
| Female, n (%) | 131 (49) | 14 (47) | 26 (48) |
| BLCLP, n (%) | 32 (12) |  | 3 (5) |
| UCLP, n (%) | 113 (42) | 4 (13) | 22 (41) |
| ICP, n (%) | 82 (31) | 26 (87) | 21 (39) |
| SMCP, n (%) | 40 (15) |  | 8 (15) |
| Age at Z-plasty, median (IQR) | 5.6 (4.4–8.1) | 5.9 (4.2–8.2) | 5.8 (4.8–8.6) |
| Syndrome  22q11.2 deletion  Van der Woude  FAS  Unknown  Goldenhar  Stickler without RS  Opitz  Asperger  6q25 microdeletion  Down  Pterygoid  Kartagener  Tourette  X-linked Siderius  Renal coloboma  Stickler with RS  Gorlin  48 XXYY syndrome  Fetal Valproate syndrome  Arnold–Chiari  Escobar  Diamond–Blackfan  Turner  De Barsy  OFCD  Cerebral palsy with PRS  Hay–Wells  Triple X  Oculodental  Congenital central hypoventilation syndrome |  |  | 6  4  4  6  3  2  2  2  2  2  2  1  1  1  1  1  1  1  1  1  1  1  1  1  1  1  1  1  1  1 |

**Pharyngeal flap (n=26)**

|  | Nonsyndromic  (n=13) | RS  (n=4) | Syndromic    (n=9) |
| --- | --- | --- | --- |
| Male, n (%) | 8 (62) | 2(50) | 6 (67) |
| Female, n (%) | 5 (38) | 2 (50) | 3 (33) |
| BLCLP, n (%) | 2 (15) |  | 2 (22) |
| UCLP, n (%) | 7 (54) | 1 (25) | 5 (56) |
| ICP, n (%) | 2 (15) | 3 (75) | 1 (11) |
| SMCP, n (%) | 2 (15) |  | 1 (11) |
| Age at velopharyngeal flap, median (IQR) | 5.1 (4.3–6.3) | 5.6 (4.9–7.8) | 6.1 (4.7–9.8) |
| Syndrome  22q11.2 deletion  Van der Woude  CHARGE, gene CHD7  Goldenhar  Asperger  48-XXYY  Kabuki  BCD  FAS  Abbreviations: Oculofasciocardiodental syndrome (OFCD), Blepharocheilodontic syndrome (BCD) and Fetal alcohol syndrome (FAS).  Bilateral cleft lip and palate (BLCLP), unilateral cleft lip and palate (UCLP), isolated cleft palate (ICP), submucous cleft palate (SMCP), Robin sequence (RS), interquartile range (IQR). |  |  | 1  1  1  1  1  1  1  1  1 |
